# Supplementary material for: Establishing charge-transfer excitons in 2D perovskite heterostructures
Source: Nat Commun. 2020 May 26;11:2618. doi: 10.1038/s41467-020-16415-1 (PMC7250833; doi:10.1038/s41467-020-16415-1)
Supplement: Supplementary file 1 — Supplementary Information [file 41467_2020_16415_MOESM1_ESM.pdf]

Supplementary Information for

Establishing charge-transfer excitons in 2D perovskite heterostructures

Zhang et al

Supplementary Figures

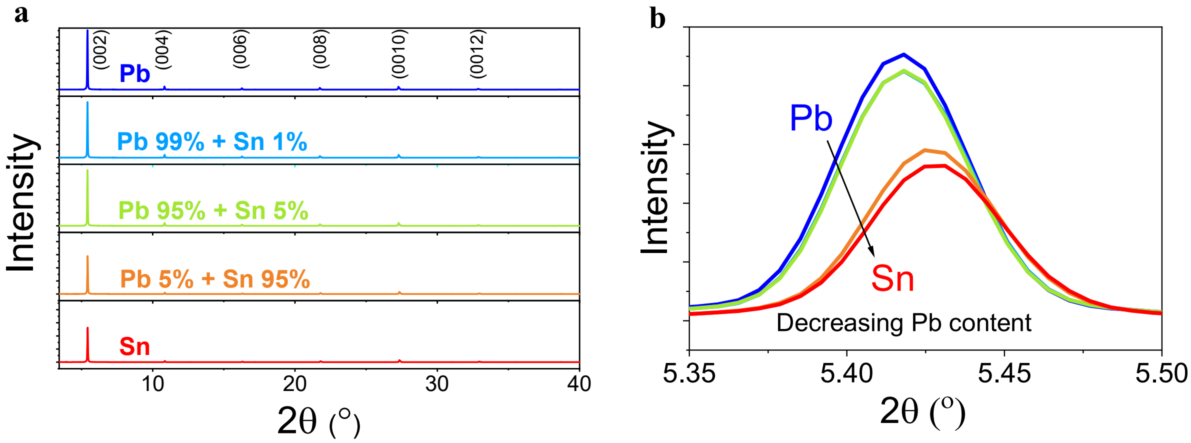

Supplementary Fig. 1 | X-ray diffraction (XRD) patterns obtained from different films. a All five films show an in-plane orientation of PbX_6 or SnI_6 sheets along substrate surface. **b** Zoom-in data to show intensities and peak positions for all five films. Blue curve: PEA_2PbI_4, sky blue curve (overlapped with green curve): $[(PEA_2PbI_4)_{0.99}$:$(PEA_2SnI_4)_{0.01}]$, green curve: $[(PEA_2PbI_4)_{0.95}$:$(PEA_2SnI_4)_{0.05}]$, orange curve: $[(PEA_2PbI_4)_{0.05}$:$(PEA_2SnI_4)_{0.95}]$ and red curve: PEA_2SnI_4.

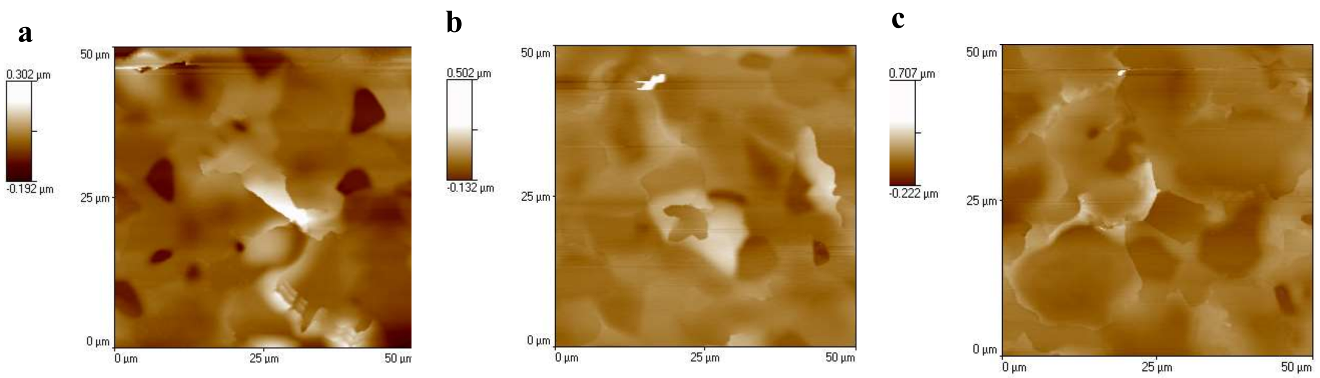

Supplementary Fig. 2 | AFM results of three different perovskite films. a PEA_2PbI_4. **b** $(PEA_2PbI_4)_{0.95}$:$(PEA_2SnI_4)_{0.05}$. **c** PEA_2SnI_4.

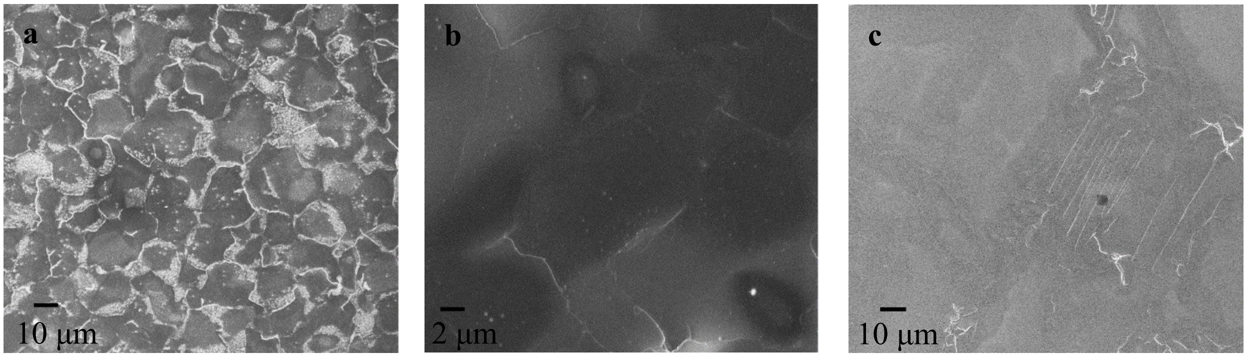

Supplementary Fig. 3 | SEM results of three different perovskite films. a PEA_2PbI_4. **b** $(PEA_2PbI_4)_{0.95}$:$(PEA_2SnI_4)_{0.05}$. **c** PEA_2SnI_4.

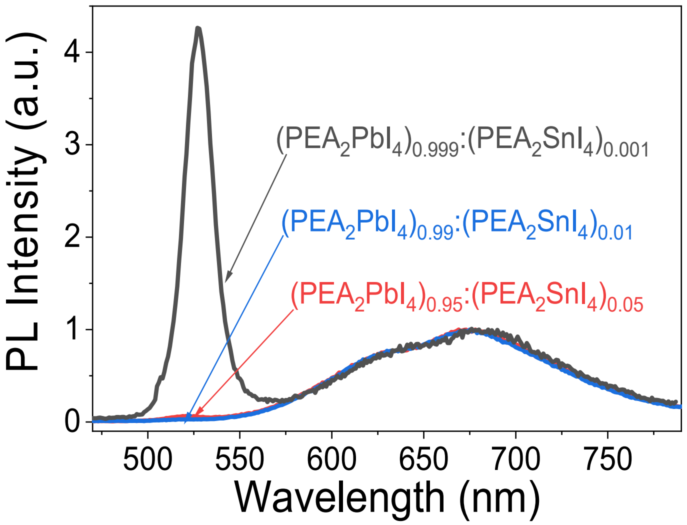

Supplementary Fig. 4 | Normalized PL spectra to show broad light emission peaked at 669 nm for heterostructured [$(PEA_2PbI_4)_{1-x}$:$(PEA_2SnI_4)_x$] films.

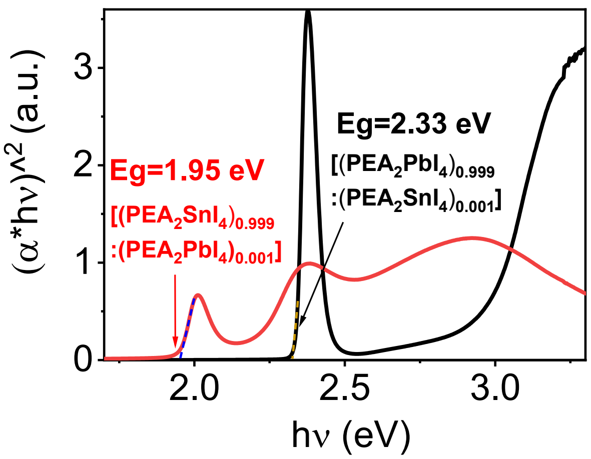

Supplementary Fig. 5 | Bandgaps determined by absorption spectra. $[(PEA_2PbI_4)_{0.9999}:(PEA_2SnI_4)_{0.001}]$ (black curve) and $[(PEA_2SnI_4)_{0.9999}:(PEA_2PbI_4)_{0.001}]$ (red curve) based heterostructured films are prepared by alternatively switching host and guest components.

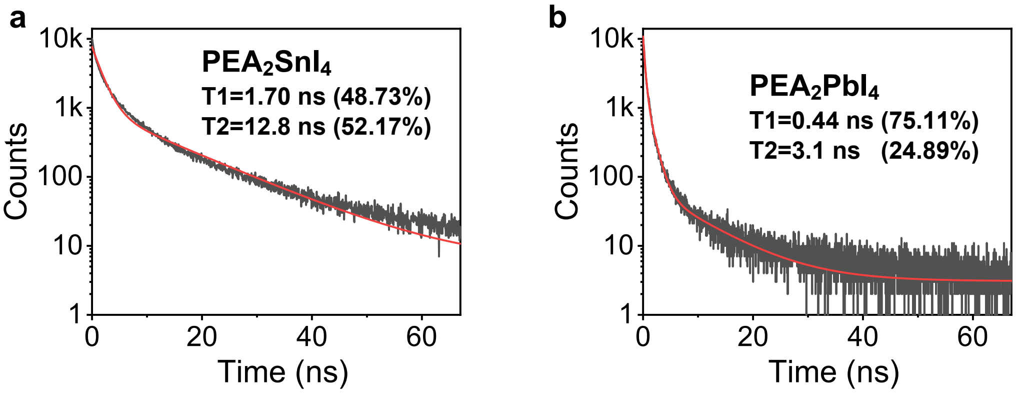

Supplementary Fig. 6 | Transient PL results for 2D Pb and Sn perovskite films. **a** Pristine PEA_2PbI_4 film. **b** Pristine PEA_2SnI_4 film.

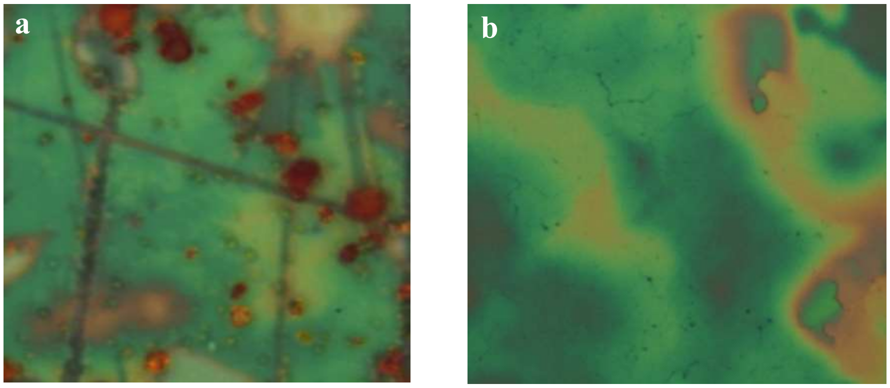

Supplementary Fig. 7 | Microscope photos under white light illumination. a Double-layered [PEA_2PbI_4/PEA_2SnI_4] film prepared by hand-finger pressing method. **b** Pure PEA_2PbI_4. The image size is 54*43μm^2.

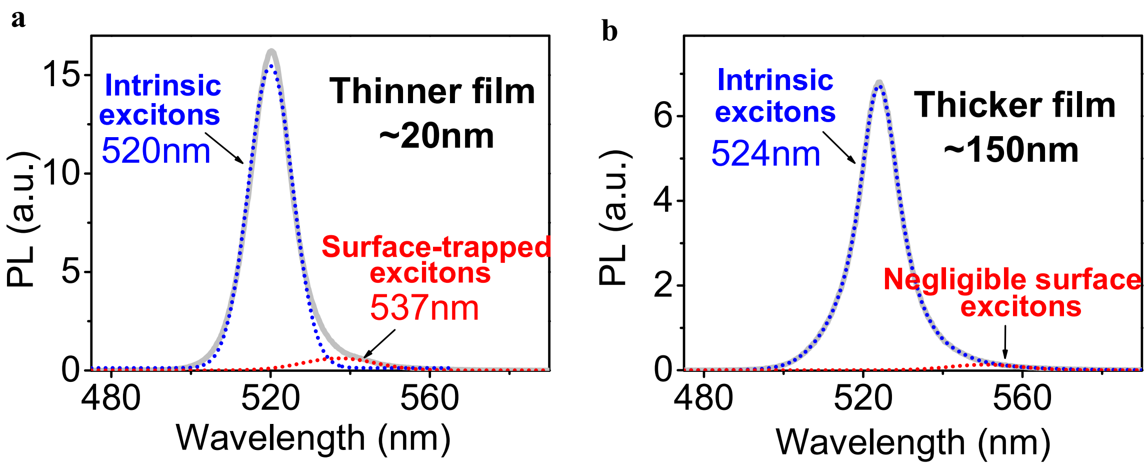

Supplementary Fig. 8 | PL spectra of pure PEA_2PbI_4 films with different thicknesses. **a** Thickness: ~20 nm; **b** Thickness: ~150 nm.

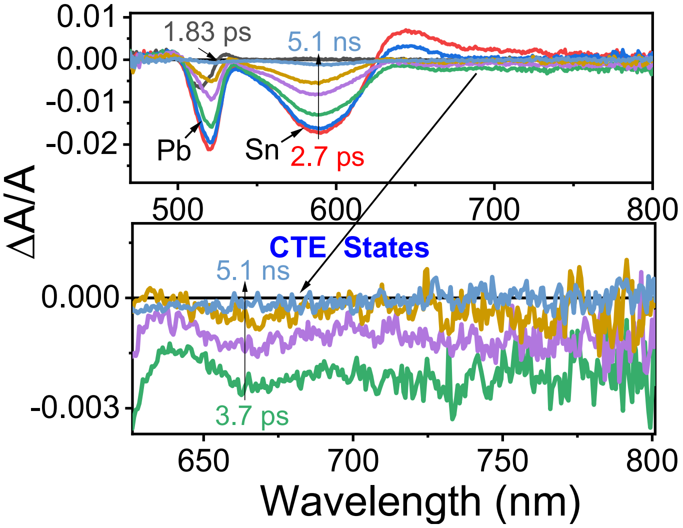

Supplementary Fig. 9 | TA spectra at different pump-delay times for double-layered PEA_2PbI_4/PEA_2SnI_4 heterointerfaces.
